# Pantoporate pollen in the Asteraceae (Vernonieae)

**DOI:** 10.3897/phytokeys.38.7495

**Published:** 2014-05-19

**Authors:** Harold Robinson, John J. Skvarla

**Affiliations:** 1 Department of Botany, MRC 166, National Museum of Natural History, P.O.Box 37012. Smithsonian Institution, Washington, DC., 20013-7012, USA; 2 Department of Botany and Microbiology, and Oklahoma Biological Survey, University of Oklahoma, Norman, Oklahoma, 73019-6131, USA

**Keywords:** Asteraceae, Compositae, *Oocephala*, pantoporate pollen, *Polydora*

## Abstract

Pantoporate pollen, which occurs sporadically in the Monocots and Dicots, has now been found in Asteraceae in two apparently related genera of the tribe Vernonieae, *Polydora* Fenzl and *Oocephala* H.Rob. Disposition of pores in *Polydora* seems more asymmetric than in *Oocephala*. Based on the known relationships within the Vernonieae, some conjectures are made regarding development of the pantoporate condition in the group.

## Introduction

The tribe Vernonieae is notable for remarkable variations in pollen structure, variations in sublophate and lophate forms with varying degrees of reduced perforated tectum in the exine and variations in the attachments of the outer exine to the footlayer. Now a new variation has been encountered during a study of South African Vernonieae that involves the first known examples of pantoporate pollen with non-equatorial pores in the Asteraceae. It is of some interest that one of the genera involved, *Polydora* (as *Crystallopollen*) was one in which pollen was first used as a taxonomic character in the Asteraceae by Steetz (1864). The new variants are occasion for some discussion of the positioning of pores in the pollen of Angiosperms.

Already known in the Tribe Vernonieae are the only examples of pollen in the Asteraceae with perforated tectum partially or completely lacking in non-colpar areas. Also unique to the tribe in the family is the six-porate form of pollen in the southeast Asian genus *Camchaya* Gagnep., where the pores occur equatorially in three pairs (Bunwong and Chantaranothai 2008, Robinson and Skvarla 2010). There is also a great deal of variation in lophate patterns in pollen of the tribe. The principle mechanism marking orientation of poles and pores and offering the most obvious provision for expansion of the grains when wet, the colpi, are lost in a set of exclusively paleotropical subtribes. As a result, many of the genera of the Vernonieae can be distinguished by the form of their pollen (Robinson and Skvarla 2010). Now, for the first time in the Vernonieae or Asteraceae we report a further variation involving pollen with pores that are pantoporate (global distribution), that is, not all equatorial in position.

## Materials and methods

Specimens examined are from the U.S. National Herbarium in Washington, D.C. Examination of pollen with a light microscope was insufficient to show the position of the pores. Pollen grains in most illustrations were treated with acetolysis (Erdtman 1960), followed by staining with osmium thiocarbohydrazide solutions and sputter coating with gold-palladium (Robinson and Skvarla 2006, 2007, Robinson et al. 2008). Unacetolyzed grains were rehydrated in water or alcohol directly from herbarium sheets and similarly sputter-coated. Observations were made with a JEOL 880 (Samuel Roberts Microscopy Laboratory at the University of Oklahoma) scanning electron microscope (SEM) or a Leica 440 (United States National Museum of Natural History in Washington scanning electron microscope, both equipped with lanthanum hexaboride (LaB_6_) electron sources. To achieve limited increased breakage, material gathered at the base of a centrifuge tube was given a few jabs with a dissecting needle.

## Results

The two genera in which the pantoporate pollen is now being reported are both restricted to sub-Saharan Africa. The two genera, *Oocephala* and *Polydora*, are evidently closely related to each other according to the pollen similarity, and both have pedunculate heads, a characteristic of many genera. Nevertheless, the two genera are easily distinguished from each other. *Polydora* has an ordinary broadly campanulate capitulum, and an ordinary capillary pappus. *Oocephala* (egghead) has an egg-shaped capitulum and a plumose pappus.

**Figure 1.**
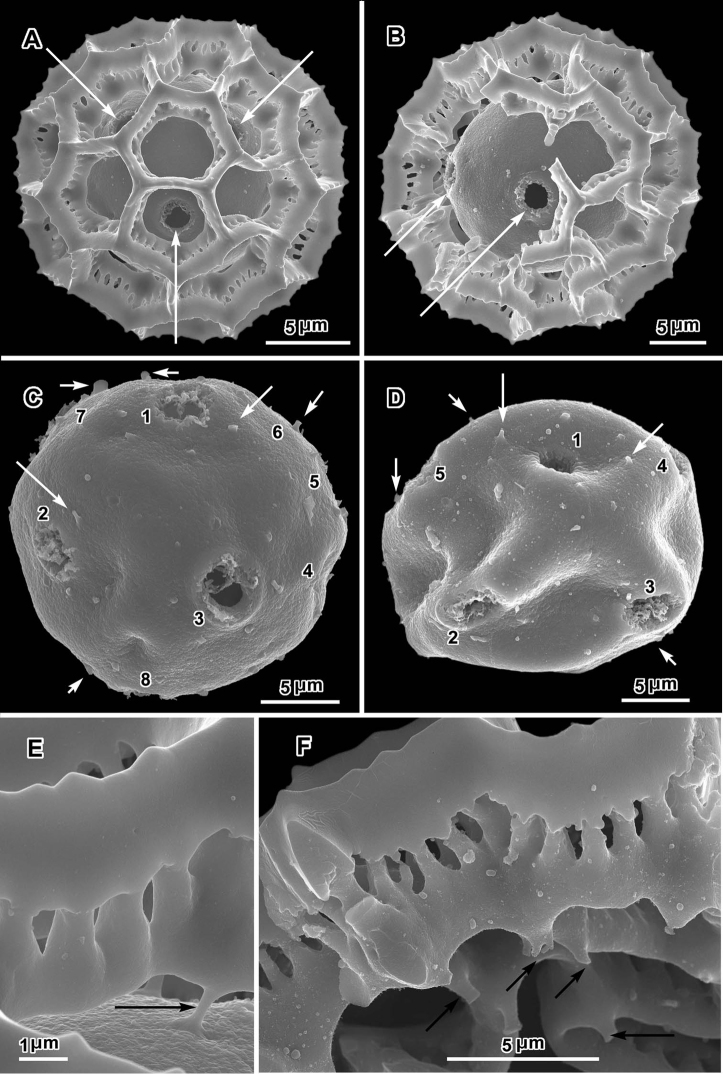
Scanning electron micrographs of *Oocephala centauroides* (*Schlechter 18138*). **A, B** pollen grains with muri of outer exine mostly ot totally intact, arrows showing visible pores **C, D** grains stripped of muri, with pores or possible pores numbered, arrows showing scars of muri attachment **D** distorted **E, F** pieces of muri showing rhizomate structure and stubs of attachments to footlayer.

**Figure 2.**
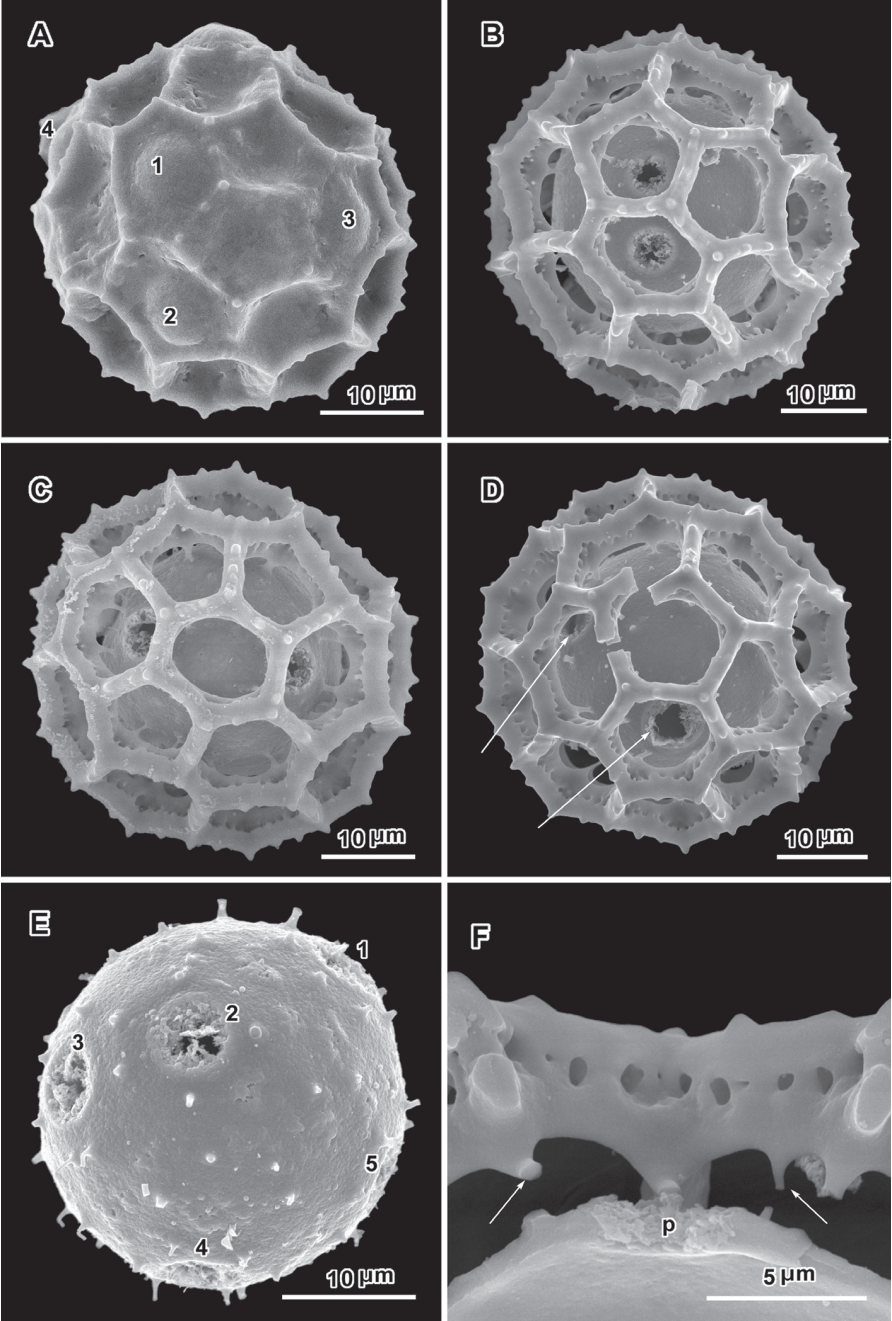
Scanning electron micrographs of *Oocephala staehelinoides* (**A–D** and **F** from *Liebenberg 8848*; E from *Liebenberg 8843*). **A** unacetylized grain showing three pores with caps intact, two pores in adjacent lacunae **B–D** intact or nearly intact grains showing pores in both pentagonal and hexagonal lacunae **B** with pores in adjacent lacunae **E** grain stripped of muri showing five pores and stubs of muri attachments **F** segment of muri showing rhizomate structure and remnants of weak basal attachments to footlayer.

**Figure 3.**
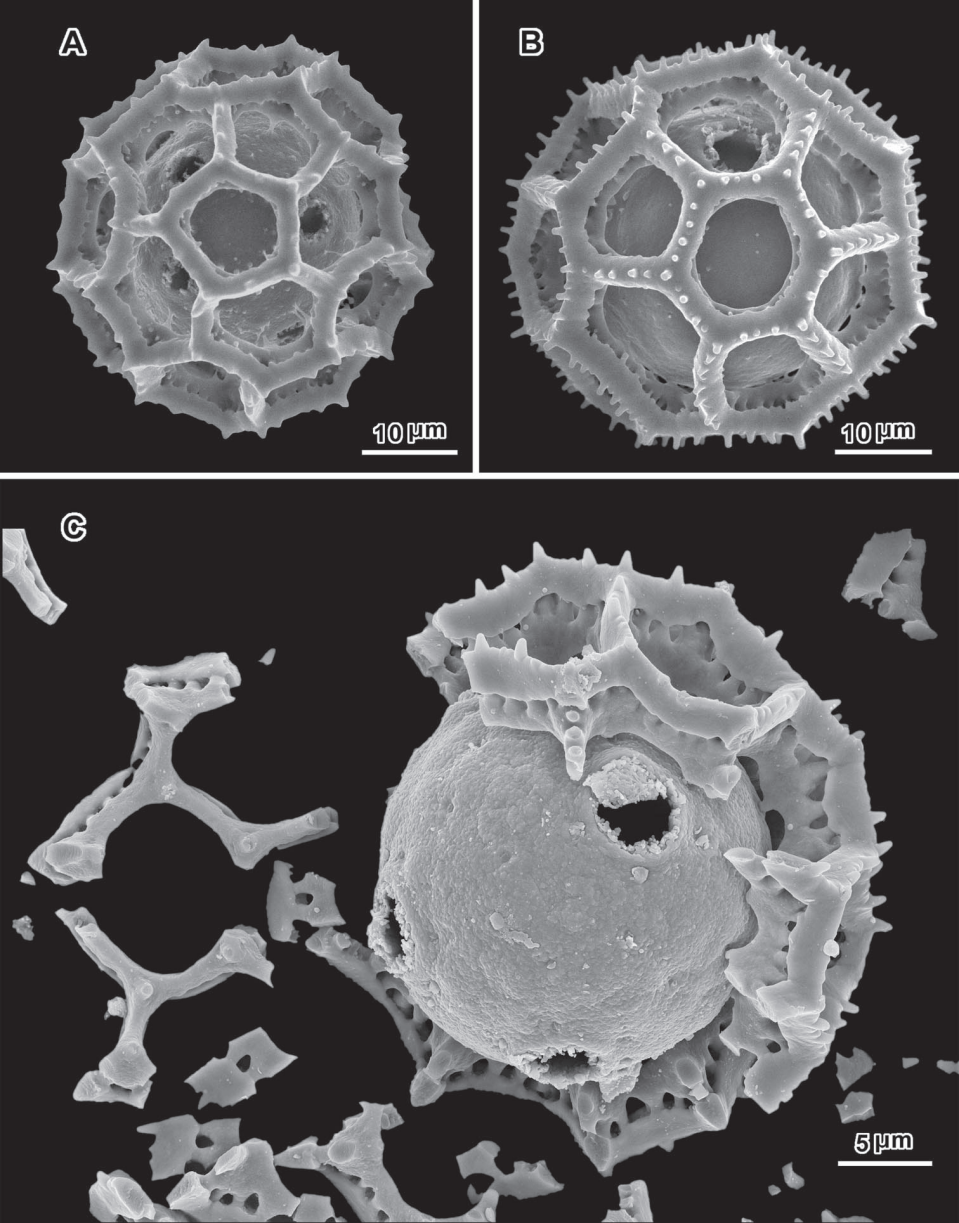
Scanning electron micrographs of *Oocephala stenocephala* (**A–B** from *Christensen & Chisumpa 1508*) and *Polydora angustifolia* (**C** from *Christensen & Patel 1457*). **A** intact grain with four visible pores **B** intact grain with one visible pore **C** grain partially stripped of muri showing three pores in pantoporate positions.

**Figure 4.**
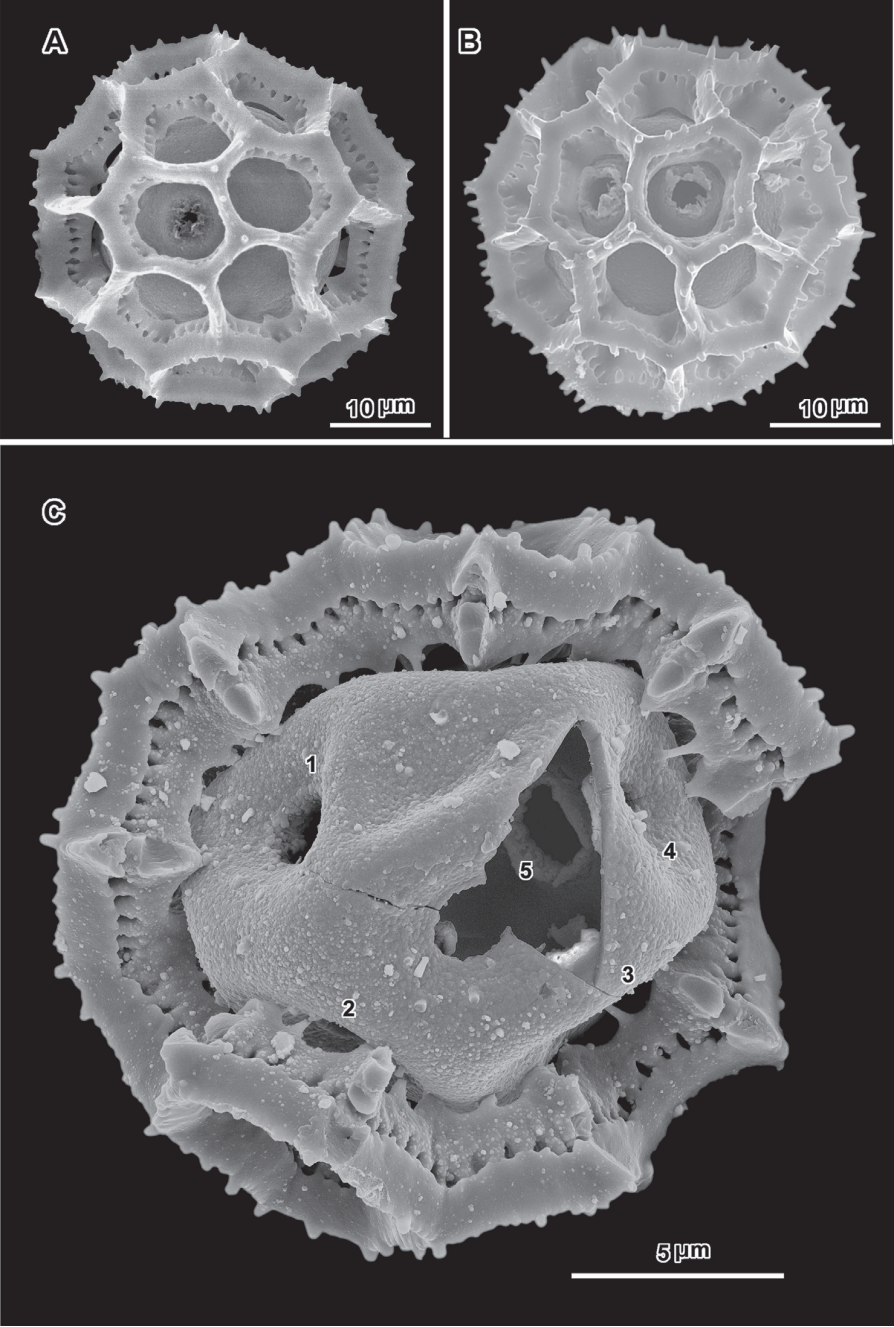
Scanning electron micrographs of *Polydora angustifolia* (**A** from *Brass 16090*; **B–C** from *Christensen & Patel 1457*). **A** intact grain with visible pore **B** intact grain with two visible pores in adjacent lacunae **C** grain with muri partially removed showing distorted inner surface and five pores.

**Figure 5.**
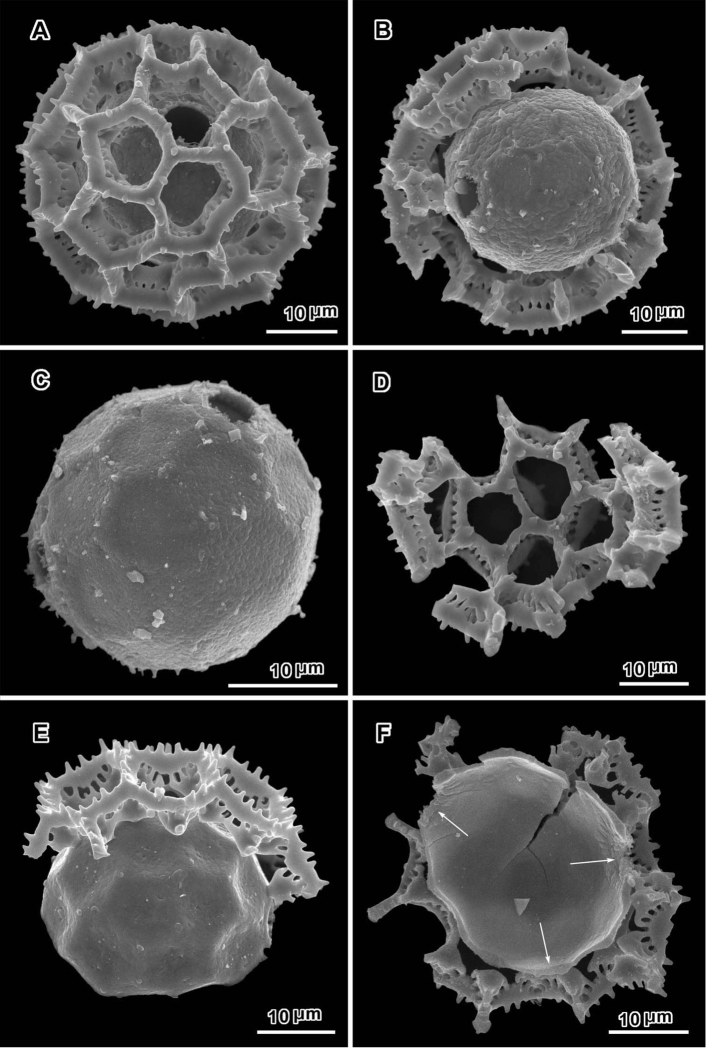
Scanning electron micrographs of *Polydora bainesii* (**A–D** from *Smith & Kaunda 4187*) and *Polydora silvicola* (**E–F** from *Christensen & Chisumpa 1526*). **A** intact grain with one visible pore **B, C** grains partially and completely stripped of muri **D** segment of outer exine showing how muri can detach in large units **E, F** broken grains **F** split grain with three pores (arrows) **B, C, E** grains showing asymmetry with large areas lacking pores.

**Figure 6.**
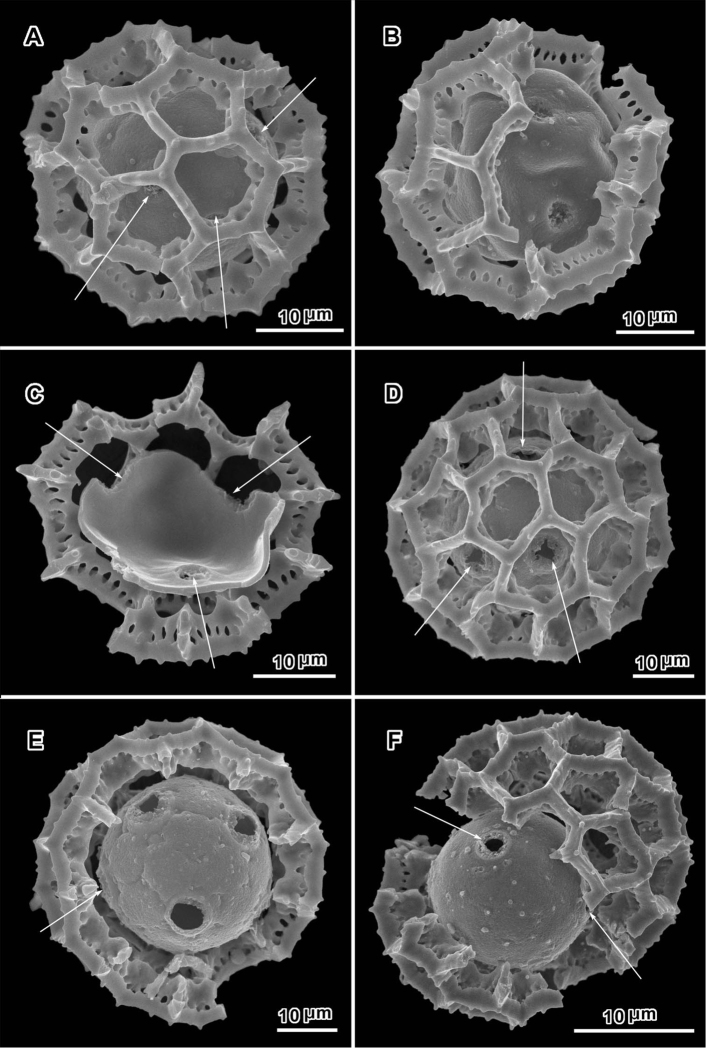
Scanning electron micrographs of *Polydora poskeana* (**A–C** from *Koekemoer 234*) and *Polydora serratuloides* (**D–F, D** and **E** from *Lualla 4640*, **F** from *Groomany 7869*). **A** intact grain with pore and incipient pores (arrows) **B** grain partially stripped of muri showing three visible pores **C** grain stripped of muri with three visible pores **D** intact grain with three visible pores, two in adjacent lacunae **E, F** broken grains with four and two pores visible.

**Figure 7.**
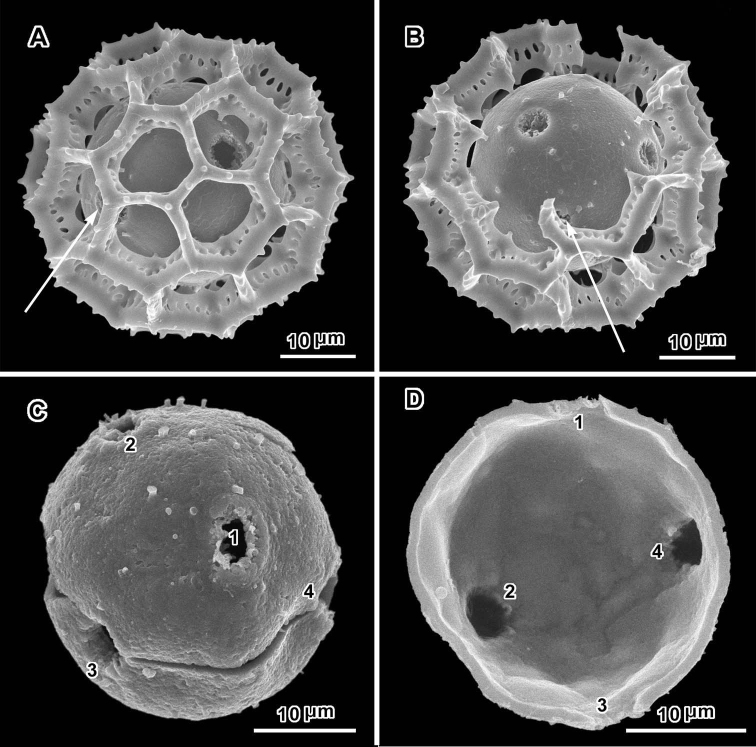
Scanning electron micrographs of *Polydora steetziana* (**A** and **B** from *Seydel 2828a*, **C** and **D** from *Koekemoer 2189*). **A** intact grain with two visible pores **B** broken grain with three visible pores **C** grain stripped of muri with four pores visible **D** broken grain with four pores visible from inside.

Examined material (Table 1) includes three species of *Oocephala*, ***Oocephala centauroides*** (Klatt) H.Rob. & Skvarla, comb. nov. [basionym: *Vernonia centauroides* Klatt, Bull. Herb. Boissier 4: 824. 1896] (Fig. 1A–F); ***Oocephala staehelinoides*** (Harv.) H.Rob. & Skvarla, comb. nov. [basionym: *Vernonia staehelinoides* Harv., Thes. Cap. 2: 36. 1863, “stahelinoides”] (Fig. 2A–F); *Oocephala stenocephala* (Oliv.) H.Rob. (Fig. 3A, B); and eight species of *Polydora*, *Polydora angustifolia* (Steetz) H.Rob. (Figs 3C, 4); *Polydora bainesii* (Oliv. & Hiern) H.Rob. (Fig. 5A–D); *Polydora chloropappa* (Baker) H. Rob; *Polydora jelfiae* (S. Moore) H.Rob.; *Polydora sylvicola* (G.V. Pope) H.Rob. (Fig. 5E, F); *Polydora poskeana* (Vatke & Hildebr.) H.Rob. (Fig. 6A–C); *Polydora steetziana* (Oliv. & Hiern) H.Rob. (Fig. 7A–C); and *Polydora serratuloides* (DC.) H.Rob. (Fig. 6D–E).

**Table 1.** Specimens studied.

433	*Oocephala centauroides* (Klatt) H.Rob. & Skvarla, Regio oriente et Mosambique, Delagoa Bay, *Schlechter 18138* (US).
474	*Oocephala staehelinoides* (Harv.) H.Rob. & Skvarla, Transvaal, Mar. 1972, *Liebenberg 8848* (US).
475	*Oocephala stenocephala* (Oliv.) H.Rob., Zambia, *Christensen & Chisumpa 1508* (US).
451	*Polydora angustifolia* (Steetz) H.Rob. (as *Polydora erinacea*), Malawi, Zomba District, Likangala, Phalombe Road, 3000 ft., 3/ 12/ 1984, *Christensen & Patel GMC 1457* (US).
461	*Polydora angustifolia* (Steetz) H.Rob., *Brass 16090* (US), isoneotype.
462	*Polydora bainesii* (Oliv. & Hiern) H.Rob., Zimbabwe, *West 7266* (US).
463	*Polydora bainesii* (Oliv. & Hiern) H.Rob., Zambia, *C. Earle Smith & Richards 4671* (US).
464	*Polydora bainesii* (Oliv. & Hiern) H.Rob., Malawi, *Salubeni & Kaunda 4187* (US).
465	*Polydora chloropappa* (Baker) H.Rob., Tanzania, *Bidgood & Vollesen 7082* (US).
466	*Polydora chloropappa* (Baker) H.Rob., Zambia, *Loveridge 845* (US).
467	*Polydora jelfiae* (Moore) H.Rob., Zambia, *Christensen & Chisumba 1512* (US).
473	*Polydora poskeana* (Vatke & Hildebr.) H.Rob., Tranvaal, *Koekemoer 234* (US).
468	*Polydora serratuloides* (DC.) H.Rob., Burundi, *Lualla 4640* (US).
469	*Polydora serratuloides* (DC.) H.Rob., Tanzania, *Groomany 7869* (US).
470	*Polydora steetziana* (Oliv. & Hiern) H.Rob., S. Africa, *Koekemoer 2189* (US).
471	*Polydora steetziana* (Oliv. & Hiern) H.Rob., Namibia, *Seydel 2828a* (US).
472	*Polydora sylvicola* (Pope) H.Rob., Zambia, *Christensen & Chisumpa GMC 1526* (US).

The pollen of both genera has a lophate exine pattern that is nearly spherically symmetrical. The pattern consists of a mixture of pentagonal and hexagonal lacunae. Perforated tectum is essentially absent leaving totally exposed muri and their subtending columellae. Both genera also share a mural structure that has been referred to as rhizomate (Robinson 1999) (Figs 1F, 2F, 3C, 4C, 5B, D, 6C, E). In these muri, the columellae, instead of being firmly attached to the footlayer at their bases, are attached basally to each other by a lower horizontal structure (rhizome) that is itself weakly attached to the footlayer. Such a structure is not unique to the two genera studied, but occurs in many lophate, triporate, non-colpate pollen grains in the subtribes Centrapalinae and Erlangeinae that are native to the Paleotropics. A note of interest is the possible difference in the chemical make-up of these muri, since they have been seen to survive the degrading effects of chloral hydrate more completely than the muri and tectum of other Vernonieae when mounted in Hoyer’s solution (Anderson 1954).

Positions of the pores are often difficult to determine in lophate pollen of the Centrapalinae and Erlangeinae. The lacunae in which the pores occur are totally undifferentiated in any other way, being either pentagonal or hexagonal, even in the polar regions. The depths of the lacunae usually prevent seeing more than one pore at a time unless the grains are unacetylized and the caps on the pores are preserved (Fig. 2A) (Robinson and Skvarla 2009). Thus, the easily dehiscent muri of *Oocephala* and *Polydora* are particularly helpful. The totally exposed sphere of the pollen footlayer shows the pores very well (Figs 1C, D, 2E, 3C, 4C, 6C, 7C, D). In this way, positions and numbers of pores on the grains can be more accurately seen (Figs 1C, D, 2E, 7C, 7D). To help in the estimates, a toy ball was obtained from the museum shops that showed a pattern of pentagons and hexagons that was a very close approximation of the pattern of lacunae in the pollen grains of *Oocephala* and *Polydora*. It was study of the toy ball that led to the conclusion that the pollen of *Oocephala* could characteristically have seven or eight pores. (Figs 1C, 2E).

An exact count of the pores can never be certain, since there is evidence of some uneven distribution of pores in some grains. In a few cases, in *Oocephala*, even in grains with the muri present, pores can be seen in adjacent lacunae (Figs 2A, B, 3A, 4B, 6D). In other cases in *Polydora* (Figs 3C–7C), expanses can be seen that have no pores (Fig. 5B, C, E). The total number of pores may never be more than the five or six that can be seen in some views of *Oocephala* (Figs 1C, D, 2E), and asymmetry is probably even more extreme in *Polydora* (Fig. 5C, E), but pantoporate non-equatorial distribution of pores is certain in both genera, and at least some asymmetry is certain.

## Discussion

Two basic porate conditions of Angiosperm pollen are well known (Walker 1974). The forms derived generally from the tetragonal or “cruciate” division of the pollen mother cell that is found in Gymnosperms, Monocots and basal Dicots. The alternative tetrahedral or decussate division of the pollen mother cell is basic to the Eudicots, a sort of reversion to the spore mother-cell pattern of division found in most Cryptogams. The polyporate (pantoporate) condition is found sporadically in all three Angiosperm groups, or as stated by Harley (2004) ”known from all three major groups of Angiosperms, although infrequently in the monocots and even less frequently in the basal dicots”. In monocots (Harley 2004, Nadot et al. 2006) It is reported in some Bromeliaceae, Alismataceae and Araceae. In basal dicots polyporate pollen is mentioned only from the Trimeniaceae (Sampson 2000, Harley 2004). In the eudicots, Harley mentions Caryophyllales, Ranunculales, Podostemaceae, Linaceae, Zygophyllaceae and Geraniaceae as achieving the polyporate condition by multiplication of simple furrows that are reduced to pores. In other families such as Malvaceae, Sterculiaceae, Thymelaeaceae, Euphorbiaceae and Malpighiaceae a spiralization of a line joining centers of apertures is suggested. All of these are in derived groups, and polyporate pollen is not basic to any group.

There have been no previous reports of pantoporate pollen in the Asteraceae, and suggestions here are based on observations of the pollen of related members of the family in the tribe Vernonieae.

The Asteraceae has pollen that is basically tricolporate, a condition that seems basic to all the tribes including the Vernonieae (Blackmore et al. 2009). The most important evidence comes from the fact that the polyporate condition in the Asteraceae is found in the tribe Vernonieae which has the some of the most destabilized pollen formation of any tribe in the family. The pantoporate pollen has developed within the subgroup of the Vernonieae that already has the most spherically symmetrical forms of pollen in the tribe, the subtribal pair, Erlangeinae and Centrapalinae. It is in these subtribes that the colpi are totally suppressed, and orderly arrangement of the lacunae, even at the poles, is completely lost. These features that have become so spherically symmetrical in these subtribes are among the last layers laid down as the pollen grain matures. The position of the pores is much more basic, being established in some of the innermost and earliest laid down layers of the pollen. It is the contention here that the loss of radial symmetry in the Vernonieae pollen started with the outermost and most belatedly deposited layers of the grains, and progressed eventually to the inner and earlier deposited layers.

There is even a developmental basis for the asymmetry in the distribution of the pores, more obvious in *Polydora* and less evident in the more specialized *Oocephala*. This could trace to the fact that the developing pollen grain has a distal surface that faces outward in the pollen mother cell, and a proximal surface that faces inward toward the center of the tetrad of pollen grains. Thus, the distribution of the pores is influenced by early stages in pollen development, beginning with position in the tetrads or mother cell.
